# Stress, Depression, Sexual Function, and Alexithymia in Infertile Females with and without Polycystic Ovary Syndrome: A Case-Control Study

**DOI:** 10.22074/ijfs.2019.5703

**Published:** 2019-07-14

**Authors:** Zahra Basirat, Mahbobeh Faramarzi, Seddigheh Esmaelzadeh, Sharareh Abedi Firoozjai, Theresa Mahouti, Zahra Geraili

**Affiliations:** 1 Infertility and Health Reproductive Research Center, Health Research Institute, Babol University of Medical Sciences, Babol, Iran; 2 Social Determinants of Health Research Center, Health Research Institute, Babol University of Medical Sciences, Babol, Iran

**Keywords:** Alexithymia, Depression, Infertilty, Polycystic Ovary Syndrome, Sexual Dysfunction

## Abstract

**Background:**

Infertile females experience some types of distress such as social stress, depression, and sexual dys- function that may be exacerbated by polycystic ovary syndrome (PCOS). The current study aimed at comparing psychological profile of infertile females with PCOS with that of women without PCOS with respect to four domains: infertility stress, depression, sexual dysfunction, and alexithymia.

**Materials and Methods:**

The current case-control study was conducted on 240 infertile females (120 with PCOS and 120 without PCOS) in Fatemeh Azahra Infertility and Reproductive Health Research Center (Babol, Iran) from 2016 to 2017. The following questionnaires were used to collect data: the fertility problem inven- tory (FPI), the female sexual function index (FSFI), the Beck depression inventory-II (BDI-II), and the Toronto alexithymia scale (TAS-20).

**Results:**

Females with PCOS had higher FPI total scores than the ones without PCOS (120.68 ± 29.42 vs. 112.83 ± 30.94). Of the subscales of infertility stress, the mean scores of social stress and rejection of a future life without a child were higher in females with PCOS than the ones without PCOS (P<0.05). Also, the mean total scores of alexithy- mia symptoms (TAS-20) in females with PCOS were significantly higher than those of the ones without PCOS (59.83 ± 11.36 vs. 55.69 ± 11.52). There was no significant difference between the two groups regarding the mean scores of depression symptoms and sexual function.

**Conclusion:**

Infertile females with PCOS experienced higher levels of infertility stress and inability to distinguish and describe their feelings compared with the ones without PCOS. It is suggested that infertility care providers should provide more psychosocial support for infertile females with PCOS.

## Introduction

Polycystic ovarian syndrome (PCOS) is one of the most common etiological factors of infertility which is identified in up to 20% of infertile females (1). Studies emphasized that the prevalence of psychiatric disorders is high in patients with PCOS. A longitudinal study reported a prevalence of 40% for depression in patients with PCOS (2). A cohort study reported that PCOS can increase the risk of schizophrenia, bipolar disorder, personality disorders, and tics (3). Other psychiatric disorders such as anxiety, eating disorders, and sexual dysfunction disorders are common in patients with PCOS (4). In addition, females with PCOS reported lower body image satisfaction compared to the ones without PCOS (5). Clinical manifestations of PCOS including menstrual irregularity, hirsutism, and acne may exacerbate distress in the affected females (6). 

Many females experience infertility as a feeling of distress and stigma (7). Infertile females experience some types of distress such as social stress, depression, sexual dysfunction, and marital dissatisfaction (8-10) that may be exacerbated by PCOS. Kitzinger and Willmott (11) introduced PCOS as a stigma, “the thief of womanhood”. Infertile females with PCOS and infertility problems may experience being less feminine, due to excessive hair growth and absence of or irregular menstrual periods (12). Additionally, infertility management processes such as assisted reproductive techniques are more stressful in females with PCOS than the ones without it (13). 

Alexithymia is a personality construct with inability in normal affect regulation that is comprised of five characteristics including difficulty to identify and distinguish emotions from bodily sensations, difficulty to describe and verbalize emotions, externally oriented thinking style, poverty of fantasy life, and poor empathy (14). This personality construct is a risk factor for various physical and mental health problems including anxiety, depression, compulsive or addictive behaviors, physical symptoms, and potentially somatic diseases (15). Since a previous study showed that infertile females had higher rates of alexithymia than the fertile ones (16), it was assumed that the rate of alexithymia may differ among infertile females with different levels of stress. Therefore, infertile females with PCOS may have different levels of alexithymia compared with the ones without PCOS.

Although many previous studies indicated that psychiatric disorders are common in patients with PCOS (2-6), few researches reported psychiatric symptoms in females with PCOS and infertility. Diamond et al. (16) concluded that female sexual dysfunction does not vary between infertile females with PCOS and the ones with unexplained infertility. Another study reported that infertility did not appear to constitute a risk factor of psychological distress in females with PCOS (17). As differences in psychological profiles between infertile females with PCOS and those without PCOS are not clear yet, the current study aimed at comparing the psychological profile of these two groups. To the authors’ best knowledge, it was the first study that compared psychological profiles of infertile females with and without PCOS in terms of four domains: infertility stress, depression, female sexual dysfunction, and alexithymia (i.e. the inability to distinguish and describe feelings and the absence of fantasies).

## Materials and Methods

### Participants

The current case-control study was conducted in Fatemeh Azahra Infertility and Reproductive Health Research Center (Babol, Iran) from May 2016 to December 2017 on 240 infertile females selected through census sampling method. The case group was composed of 120 females with a definite diagnosis of PCOS. The control group was comprised of 120 infertile females without PCOS based on Rotterdam diagnostic criteria. Besides, the control group was matched with the case group in terms of age, level of education, and duration of infertility.

Inclusion criteria for infertile females with and without PCOS were being 15-45 years old, completion of primary school as the minimum level of education, being married and having an active sex life, and lacking any problems in speaking or understanding the Persian language; also, a definite diagnosis of PCOS was an additional criterion for PCOS group. Definite diagnosis of PCOS was done based on two of the following Rotterdam diagnostic criteria: ultrasound scan of PCOS (presence of .12 follicles in one or both ovaries and/ or increased ovarian volume >10 mL), clinical signs of hyperandrogenism (hirsutism or obvious acne), and/or an elevated plasma testosterone level, and/or irregular menstrual periods (interval between menstrual periods >35 days, amenorrhea defined as the absence of vaginal bleeding for .6 months, and/or variable menstruation) (18, 19).

Exclusion criteria for all participants (females with and without PCOS) were diagnosis of the husband with azoospermia or oligospermia, presence of other disorders that could mimic PCOS syndrome such as congenital adrenal hyperplasia, thyroid disease, or hyperprolactinemia.

### Procedure

Four staff of the infertility center explained the study’s objectives to the participants and accordingly, the subjects were required to sign the written informed consent forms. The staff interviewed the subjects and recorded their demographic characteristics, as well as their medical and gynecological history. Furthermore, the subjects were asked to complete five questionnaires of the study including the fertility problem inventory (FPI), the female sexual function index (FSFI), the Beck depression inventory-II (BDI-II), and the Toronto alexithymia scale (TAS-20). First, 258 females (129 with and 129 without PCOS) were enrolled of which 240 females with infertility (120 with and 120 without PCOS) completed the questionnaires. 

### Ethical considerations

The current study was approved by the Ethics Committee of Babol University of Medical Sciences (No.4834).

### Measures 

#### Demographic questionnaire

Demographic characteristics including age, educational level, infertility history, clinical information of PCOS, and assisted reproductive technology (ART) history were obtained from the subjects. In addition, weight and height were measured in order to obtain body mass index (BMI).

#### Infertility stress

Infertility stress was assessed using FPI developed by Newton in 1999. It is a multi-dimensional tool to detect stress and infertility problems. The FPI is comprised of 46 questions divided in five subscales: social concern, sexual concern, relationship concern, rejection of parenthood, and the need for parenthood. Each item is scored based on a six-point Likert scale, ranging from 1 (strongly disagree) to 6 (strongly agree). The total score ranges from 46 to 276 with higher scores representing higher levels of stress (20). Validity and reliability of the Persian version of FPI were previously examined (21). In the current study, the Cronbach’s alpha coefficient of the FPI was 0.898.

### Sexual function

The FSFI was used to assess sexual function in subjects. The FSFI assesses sexual function over the past four weeks. It covers six domains: desire, arousal, lubrication, orgasm, satisfaction, and pain. The score for each domain ranges from 0 or 1 to 5 with higher scores representing better sexual function (22). It was previously shown that the Persian version of FSFI has high validity and reliability (23). In the current study, the Cronbach’s alpha coefficient of the FSFI was 0.896.

### Depression symptoms

Depression was measured by the BDI-II. It is a self- reported scale and a screening instrument for depression with 21 items, most of which assess depressive symptoms on a four-point Likert scale ranging from 0 to 3. Total scores range from 0 to 63. In clinical settings, the severity of depression based on BDI-II, is classified as follows: 0-13: minimal depression; 14-19: mild depression; 20- 28: moderate depression; and 29-63: severe depression (24). A valid Persian version of the BID-II was used in the current study. The internal consistency (Cronbach’s alpha=0.87) and test re-test reliability (r=0.74) of the BID-II Persian was high and acceptable (25). In the current study, the Cronbach’s alpha coefficient of the BDI-II was 0.915.

### Alexithymia

In the current study, alexithymia was assessed using TAS-20. It is one of the most common instruments to measure alexithymia that has 20 items in three subscales: difficulty to describe emotions, difficulty to identify feelings (DIF), and externally-oriented thinking. Items are scored based on a five-point Likert scale, ranging from 1 (strongly disagree) to 5 (strongly agree). The total alexithymia score ranges from 20 to 100 (26). A study conducted by Besharat supported the internal consistency, test-retest reliability, and concurrent validity of the Persian version of TAS-20 (27). In the current study, the Cronbach’s alpha coefficient of the TAS-20 was 0.809.

### Statistical analysis

All data were analyzed using SPSS for Windows, version 18.0 (SPSS Inc., Chicago, IL, USA). To present characteristics of females with and without PCOS, continuous variables are expressed as mean ± SD and categorical variables as numbers (%). Chi-square test was employed to compare categorical variables such as educational attainment level, duration of infertility, regularity of menstruation, and BMI between the two groups. Also, independent samples t test was employed to compare the means of age and duration of marriage between the two groups. In addition, comparisons of the mean scores between females with PCOS and those without PCOS in all four questionnaires and their subscales including FPI, FSFI, TAS-20, and BDI-II, were done using independent t test. A P<0.0.5 was considered statistically significant.

## Results

Table 1 provides the summarized demographic information of subjects in the two groups. There were no significant differences between the two groups regarding the subjects’ age, husbands’ age, educational level of the subjects, educational level of their husbands, and duration of infertility (P>0.05 in all cases). The frequency of irregular menstruation was significantly higher in females with PCOS than the ones without PCOS (P<0.001). 

**Table 1:** Demographic characteristics of women with and without polycystic ovary syndrome (PCOS)


Variable	Yes (n=120)	No (n=120)	P value

Age (Y)	29.55 ± 5.17	29.33 ± 6.23	0.771
Education			0.278
≤12 years	51 (56.7)	39 (43.3)	
>12 years	63 (49.2)	65 (50.8)	
BMI			0.218
<25	41 (34.2)	29 (24.2)	
25-29.99	45 (37.5)	52 (43.3)	
≥30	34 (28.3)	39 (32.5)	
Duration of infertility (Y)			0.159
<5	66 (74.2)	56 (64.4)	
≥5	23 (25.8)	31 (35.64)	
Regular menstruation			<0.001
Regular	64 (53.3)	93 (77.5)	
Irregular	56 (47.7)	27 (22.5)	
Duration of marriage (Y)	5.9 ± 3.99(5)	6.04 ± 3.88(5)	0.587
Husband’ age (Y)	33.06 ± 5.43	32.66 ± 4.82	0.554
Husbands’ education^*^			0.504
≤12 years	54 (47.8)	45 (43.3)	
>12 years	59 (52.2)	59 (56.7)	


Data are presented as mean ± SD or n (%). BMI; Body mass index, *; There were some missing data; therefore, the sum of the frequencies for qualitative variables is not equal to 120.

Table 2 a comparison in the mean scores of FPI, FSFI, BDI-II, and TAS-20 between the two groups. The results of the t-test revealed that females with PCOS had higher total mean scores of infertility stress (FPI) than the ones without PCOS (120.68 ± 29.42 vs. 112.83 ± 30.94, P=0.046).

**Table 2:** Comparison of psychological profile of women with and without polycystic ovary syndrome (PCOS)


Variable	PCOS		P value
		Yes (n=120)		No (n=120)	

Infertility stress (FPI)			
Social concerns	24.20 ± 8.31	21.74 ± 8.39	0.024
Sexual concerns	17.53 ± 7.98	16.75 ± 7.81	0.455
Marital concerns	25.15 ± 7.25	24.30 ± 7.34	0.371
Acceptance of life without child	18.57 ± 7.28	16.27 ± 7.87	0.021
Need for parenthood	36.06 ± 9.56	35.15 ± 9.67	0.467
Total scores	120.68 ± 29.42	112.83 ± 30.94	0.046
Alexithymia (TAS-20)			
Difficulty in describing feelings	15.17 ± 4.08	13.94 ± 3.62	0.015
Difficulty in identifying feelings	22.62 ± 6.06	19.74 ± 6.03	<0.001
Externally-oriented thinking	22.04 ± 4.26	22.38 ± 4.03	0.532
Total scores	59.83 ± 11.36	55.69 ± 11.52	0.005
Sexual dysfunction (FSFI)			
Desire	3.94 ± 0.85	3.92 ± 0.84	0.835
Orgasm	3.5 ± 0.8	3.49 ± 0.84	0.975
Satisfaction	4.78 ± 1.19	4.92 ± 1.05	0.364
Pain	4.64 ± 1.13	4.80 ± 1.16	0.279
Arousal	3.92 ± 0.92	3.88 ± 0.91	0.752
Lubrication	4.41 ± 0.85	4.49 ± 0.73	0.476
Total scores	25.13 ± 3.95	25.35 ± 3.87	0.660
Depression symptoms (BDI-II)	18.06 ± 12.03	15.65±11.76	0.121
Severity of depression			0.114
Minimum	31 (26.1)	46 (39.0)	
Mild	35 (29.4)	27 (22.9)	
Moderate	33 (27.7)	33 (28.0)	
Severe	20 (16.8)	12 (10.1)	


Ranges scores; social concern (1-60), sexual concern (1-48), relationship concern (1-60), rejection of life without child (1-48), need for parenthood (1-60), total scores of infertility stress (46-276). Difficulty in describing emotions (1-25), Difficulty in identifying feeling (1-35), Externally-oriented thinking (1-40), total scores of alexithymia (20-100). Desire (6-0), arousal (6-0), lubrication (6-0), orgasm (6-0), satisfaction (6-0), pain (6-0), total scores of sexual dysfunction (36-0). Depression symptoms (0-63), Minimum (0-13), mild (14-19), moderate (20-28), severe (29-63). Data are presented as mean ± SD or n (%).

Of the subscales of infertility stress, the mean scores of social stress and rejection of life without child were higher in females with PCOS than those of the other group (P=0.024 and P=0.021, respectively). There were no significant differences in the mean scores of subscales of sexual stress, marital stress, and parental stress between the two groups. Also, females with PCOS had higher total mean scores of alexithymia symptoms (TAS-20) than the ones without PCOS (59.83 ± 11.36 vs. 55.69 ± 11.52, P=0.005). Of the subscales of TAS-20, DIF and difficulty to describe feeling were significantly higher in females with PCOS than the ones in the other group (P<0.001 and P=0.015, respectively). There was no significant difference between the two groups in the mean scores of depression symptoms. In addition, severity of depressive symptoms did not significantly differ between the two groups (18.06 ± 12.03 vs. 15.65 ± 11.76, P=0.121). Total scores of FSFI and all its six subscales did not significantly differ between the two groups (25.13 ± 3.95 vs. 25.35 ± 3.87, P=0.660).

## Discussion

The current study aimed at comparing the psychological profiles of infertile females with PCOS with those of women without PCOS. The results showed that females with PCOS had higher total mean scores of infertility stress (FPI) than the ones without PCOS. Infertile females with PCOS had more social concerns than the ones in the other group. Also, infertile females with PCOS had more stress of rejection of life without child than the other group. To the authors' best knowledge, no published study has examined various aspects of infertility stress in infertile females with and without PCOS. However, some studies evaluated social relationships in patients with PCOS compared to the controls. Such studies reported that the social relationships of patients with PCOS was more impaired compared to the normal population (28, 29). A recent study on the development of specific measurements of quality of life in patients with PCOS emphasized on the negative effects of PCOS on family and friends and social relationships (30). Consistently, another study reported that the majority of females with PCOS (76.1%) worried about their future life without any children (15).

Now, higher intensity of infertility stress observed in infertile females with PCOS compared to the ones without PCOS, should be explained. There are some hypotheses to explain this finding. First, the secondary analysis of the data showed that symptoms of PCOS such as obesity and hirsutism were related to infertility stress. Second, some previous studies confirmed that females with PCOS experienced social pressure due to hirsutism, especially excessive facial hair (31). A study showed that hirsutism score of females with PCOS was significantly correlated with mental health status (32). Infertile females with PCOS that had hirsutism felt “unfeminine” and “different” (33). Therefore, social concerns of infertile females with PCOS may be more than those of the ones without PCOS.

In contrast with the current study’s expectation, the total scores of FSFI and all of its six subscales did not significantly differ between females with PCOS and those without PCOS. Results of some previous studies were consistent with the findings of the present study reporting that females with PCOS did not have more depression symptoms than the ones without PCOS (34, 35). However, a study reported that infertile females with PCOS had significantly higher depression scores compared to the ones without PCOS (36). A study reported that females with PCOS with ones with no desire for a child did not show significant differences in specific aspects of sexual satisfaction compared to the ones with no desire for a child (17). A recent study reported no significant difference in female sexual dysfunction disorders between infertile females with PCOS and those without PCOS (16).

The current study also aimed at comparing the alexithymia between infertile females with and those without PCOS. The results of the current study indicated that infertile females with PCOS had higher alexithymia scores than the ones without PCOS. Infertile females with PCOS had more difficulty to identify their feelings and describe their emotions compared to the ones without PCOS. To the authors’ best knowledge, no previous study assessed the alexithymia in infertile females with PCOS. Although the current study did not have enough information about the reasons for higher alexithymia in females with PCOS compared to the ones without PCOS, several hypotheses could be proposed. First, there are associations between alexithymia and maladaptation to stress. A study investigated the association between alexithymia and fertility- related stress in females with infertility demonstrated that alexithymia was related to fertility-related stress. The authors concluded that alexithymia acted as a secondary coping strategy in females with infertility (37). Second, alexithymia is related to somatization disorders. A recent meta-analysis reported that females with PCOS were more likely to have higher somatization disorders compared to the ones without PCOS (38). Third, another study introduced alexithymia and somatization as the consequences of maladaptation to stress of infertility (39). Therefore, it is supposed that high somatization in females with PCOS and infertility was comorbid with alexithymia and higher infertility stress than ones without PCOS.

Due to some limitations of the current study, data should be interpreted with caution. First, the case-control nature of the current study prevents drawing any conclusions concerning possible relationships. Prospective cohort studies in the area using reliable approaches are required to describe the casual relationship between infertile females with PCOS and those without PCOS. Second, data was collected using self-report scales that may result in underreporting of the conditions. Future studies using more reliable methods such as interviewing, might give a better picture of the psychological profile of infertile females with PCOS. Third, all of the patients included in the current study were recruited from one hospital, rather than multiple centers, that could be a limitation of the current study. Fourth, the study sample was small and cannot be generalized to numerous phenotypes of PCOS. Further, multi-centered studies with larger sample sizes are recommended. Finally, since the study was the first work that showed higher alexithymia in infertile females with PCOS, more studies in the area should investigate the extent of the associations between alexithymia and PCOS in females with infertility. Additionally, future studies are required to clarify how alexithymia arises in infertile females with PCOS.

## Conclusion

The current study results showed that infertile females with PCOS experience more infertility stress than the ones without PCOS. Also, infertile females with PCOS had higher means of alexithymia, especially with respect to the ability to distinguish and describe, compared to the ones without PCOS. The results of the current study indicated that infertility care providers should provide more psychosocial support for infertile females with PCOS. The current study was a step to present the profiles of infertile females with PCOS; thus, further longitudinal studies are required to follow the changes in psychological profiles of females with and without PCOS during infertility treatment.

## References

[1] Badawy A, Elnashar A (2011). Treatment options for polycystic ovary syndrome. Int J Womens Health.

[2] Kerchner A, Lester W, Stuart SP, Dokras A (2009). Risk of depression and other mental health disorders in women with polycystic ovary syndrome: a longitudinal study. Fertil Steril.

[3] Cesta CE, Månsson M, Palm C, Lichtenstein P, Iliadou AN, Landén M (2016). Polycystic ovary syndrome and psychiatric disorders: Co-morbidity and heritability in a nationwide Swedish cohort. Psychoneuroendocrinology.

[4] Rassi A, Veras AB, dos Reis M, Pastore DL, Bruno LM, Bruno RV (2010). Prevalence of psychiatric disorders in patients with polycystic ovary syndrome. Compr Psychiatry.

[5] Himelein MJ, Thatcher SS (2006). Depression and body image among women with polycystic ovary syndrome. J Health Psychol.

[6] Benson S, Hahn S, Tan S, Mann K, Janssen OE, Schedlowski M (2009). Prevalence and implications of anxiety in polycystic ovary syndrome: results of an internet-based survey in Germany. Hum Reprod.

[7] Daly K (1988). Reshaped parenthood identity: the transition to adoptive parenthood. J Contemp Ethnogr.

[8] Vittengl JR, Jarrett RB, Weitz E, Hollon SD, Twisk J, Cristea I (2016). Divergent outcomes in cognitive behavioral therapy and pharmacotherapy for adult depression. Am J Psychiatry.

[9] Pasha H, Basirat Z, Esmailzadeh S, Faramarzi M, Adibrad H (2017). Marital intimacy and predictive factors among infertile women in northern Iran. J Clin Diagn Res.

[10] Pasha H, Faramarzi M, Esmailzadeh S, Kheirkhah F, Salmalian H (2013). Comparison of pharmacological and nonpharmacological treatment strategies in promotion of infertility self-efficacy scale in infertile women: a randomized controlled trial. Iran J Reprod Med.

[11] Kitzinger C, Willmott J (2002). The thief of womanhood: women's experience of polycystic ovarian syndrome. Soc Sci Med.

[12] Costello MF, Ledger WL (2012). Evidence-based management of infertility in women with polycystic ovary syndrome using surgery or assisted reproductive technology. Women's Health (Lond).

[13] Taylor GJ (1987). Psychosomatic medicine and contemporary psychoanalysis.

[14] Lumely MA, Neely LC, Burger AJ (2007). The assessment of alexithymia in medical settings: implications for understanding and treating health problems. J Pers Assess.

[15] Lamas C, Chambry J, Nicolas I, Frydman R, Jeammet P, Corcos M (2006). Alexithymia in infertile women. J Psychosom Obstet Gynecol.

[16] Diamond MP, Legro RS, Coutifaris C, Alvero R, Robinson RD, Casson PA (2017). Sexual function in infertile women with polycystic ovary syndrome and unexplained infertility. Am J Obstet Gynecol.

[17] Tan S, Hahn S, Benson S, Janssen OE, Dietz T, Kimmig R (2008). Psychological implications of infertility in women with polycystic ovary syndrome. Hum Reprod.

[18] Rotterdam ESHRE/ASRM-Sponsored PCOS Consensus Workshop Group (2004). Revised 2003 consensus on diagnostic criteria and long-term health risks related to polycystic ovary syndrome. Fertil Steril.

[19] Esmailzadeh S, Faramarzi M, Jorsarai G (2005). Comparison of in vitro fertilization outcome in women with and without sonographic evidence of polycystic ovarian morphology. Eur J Obstet Gynecol Reprod Biol.

[20] Newton CR, Sherrard W, Glavac I (1999). The Fertility Problem Inventory: measuring perceived infertility-related stress. Fertil Steril.

[21] Omani Samani R, Almasi-Hashiani A, Shokri F, Maroufizadeh S, Vesali S, Sepidarkish M (2017). Validation study of the Fertility Problem Inventory in Iranian infertile patients. Middle East Fertil Soc J.

[22] Rosen R, Brown C, Heiman J, Leiblum S, Meston C, Shabsigh R (2000). The female sexual function index (FSFI): a multidimensional self-report instrument for the assessment of female sexual function. J Sex Marital Ther.

[23] Mohammdi KH, Heydari M, Faghihzadeh S (2008). The female sexual function index (FSFI): validation of the Iranian version. Payesh.

[24] Beck AT, Steer RA, Garbin MG (1988). Psychometric properties of the beck depressive inventory: twenty-five years of evaluation. Clin Psychol Rev.

[25] Ghassemzadeh H, Mojtabai R, Karamghadiri N, Ebrahimkhani N (2005). Psychometric properties of a Persian-language version of the beck depression inventory--second edition: BDI-II-Persian. Depress Anxiety.

[26] Bagby RM, Parker JD, Taylor GJ (1994). The twenty-item toronto alexithymia scale--I.Item selection and cross-validation of the factor structure. J Psychosom Res.

[27] Besharat MA (2007). Reliability and factorial validity of a Farsi version of the 20-item Toronto alexithymia scale with a sample of Iranian students. Psychol Rep.

[28] Rzońca E, Bień A, Wdowiak A, Szymański R, Iwanowicz-Palus G (2018). Determinants of quality of life and satisfaction with life in women with polycystic ovary syndrome. Int J Environ Res Public Health.

[29] Costa EC, DE Sá JCF, Stepto NK, Costa IBB, Farias-Junior LF, Moreira SDNT (2018). Aerobic training improves quality of life in women with polycystic ovary syndrome. Med Sci Sports Exerc.

[30] Williams S, Sheffield D, Knibb RC (2018). The Polycystic Ovary Syndrome Quality of Life scale (PCOSQOL): development and preliminary validation. Health Psychol Open.

[31] Coffey S, Mason H (2003). The effect of polycystic ovary syndrome on health-related quality of life. Gynecol Endocrinol.

[32] Hahn S, Janssen OE, Tan S, Pleger K, Mann K, Schedlowski M (2005). Clinical and psychological correlates of quality-of-life in polycystic ovary syndrome. Eur J Endocrinol.

[33] Elsenbruch S, Hahn S, Kowalsky D, Offner AH, Schedlowski M, Mann K (2003). Quality of life, psychosocial well-being, and sexual satisfaction in women with polycystic ovary syndrome. J Clin Endocrinol Metab.

[34] Hahn S, Janssen OE, Tan S, Pleger K, Mann K, Schedlowski M (2005). Clinical and psychological correlates of quality-of-life in polycystic ovary syndrome. Eur J Endocrinol.

[35] Himelein MJ, Thatcher SS (2006). Depression and body image among women with polycystic ovary syndrome. J Health Psychol.

[36] Gourounti K, Anagnostopoulos F, Griva F, Vaslamatzis G (2016). Alexithymia and fertility-related stress. Women Health.

[37] Brutocao C, Zaiem F, Alsawas M, Morrow AS, Murad MH, Javed A (2018). Psychiatric disorders in women with polycystic ovary syndrome: a systematic review and meta-analysis. Endocrine.

[38] Conrad R, Schilling G, Hagemann T, Haidl G, Liedtke R (2003). Somatization and alexithymia in male infertility.A replication study. Hautarzt.

[39] Lanigan S, Kwan C, Dykes P, Gonzales M (2000). Quality of life studies in hirsute women receiving ruby laser treatment. Br J Dermatol.

